# Genome-Wide Functional and Stress Response Profiling Reveals Toxic Mechanism and Genes Required for Tolerance to Benzo[a]pyrene in *S. cerevisiae*

**DOI:** 10.3389/fgene.2012.00316

**Published:** 2013-02-08

**Authors:** Sean Timothy Francis O’Connor, Jiaqi Lan, Matthew North, Alexandre Loguinov, Luoping Zhang, Martyn T. Smith, April Z. Gu, Chris Vulpe

**Affiliations:** ^1^Vulpe Laboratory, Graduate Group in Environmental Health Sciences, University of CaliforniaBerkeley, CA, USA; ^2^Gu Laboratory, Department of Civil and Environmental Engineering, Northeastern UniversityBoston, MA, USA; ^3^Vulpe Laboratory, Department of Nutritional Science and Toxicology, University of CaliforniaBerkeley, CA, USA; ^4^Genes and Environmental Laboratory, Division of Environmental Health Sciences, School of Public Health, University of CaliforniaBerkeley, CA, USA

**Keywords:** benzo[a]pyrene, toxicity, yeast, stress, resistance, sensitivity, S-9, ontology

## Abstract

Benzo[a]pyrene (BaP) is a ubiquitous, potent, and complete carcinogen resulting from incomplete organic combustion. BaP can form DNA adducts but other mechanisms may play a role in toxicity. We used a functional toxicology approach in *S. cerevisiae* to assess the genetic requirements for cellular resistance to BaP. In addition, we examined translational activities of key genes involved in various stress response pathways. We identified multiple genes and processes involved in modulating BaP toxicity in yeast which support DNA damage as a primary mechanism of toxicity, but also identify other potential toxicity pathways. Gene ontology enrichment analysis indicated that DNA damage and repair as well as redox homeostasis and oxidative stress are key processes in cellular response to BaP suggesting a similar mode of action of BaP in yeast and mammals. Interestingly, toxicant export is also implicated as a potential novel modulator of cellular susceptibility. In particular, we identified several transporters with human orthologs (solute carrier family 22) which may play a role in mammalian systems.

## Introduction

Benzo[a]pyrene (BaP) is a potent, ubiquitous, and persistent animal carcinogen as well as a probable human carcinogen (International Agency for Research on Cancer class I carcinogen) produced by incomplete organic matter combustion. It readily distributes to soil and dust particles (Hattemer-Frey and Travis, 1991; Chung et al., 2011). The major routes of exposure are food, both due to endogenous contamination and cooking chemistry, as well as ambient air (indoor and outdoor) where vehicular exhaust and traditional combustion cooking and heating processes are the main source of BaP exposure aside from smoking (Mitchell, 1982; Wester et al., 1990; Laurent et al., 2002; Roseiro et al., 2011). Inhalation is the most effective route of BaP absorption, followed by ingestion, and then dermal uptake; however, the largest exposure is likely by ingestion (Wester et al., 1990; Hattemer-Frey and Travis, 1991; Edoardo, 1992; Laurent et al., 2002; Fromme et al., 2004; Moody et al., 2007).

Once in the body, BaP preferentially distributes to the kidneys and liver, with lower levels partitioning to the testes, brain, and spleen (Mitchell, 1982). BaP is difficult to excrete owing in part to its affinity for adipose tissues and its interaction with serum proteins, both of which can cause the chemical to remain in the body for days despite metabolic action (Shu and Bymun, 1983). BaP is metabolized by cytochrome P-450 enzymes (CYPs) to more excretable yet highly reactive and toxic metabolites (Baird et al., 2005). One of these, benzo[a]pyren-7,8-dihydrodiol-9,10-epoxide (BPDE) is thought to form DNA adducts and facilitate the induction of cancer via guanine to thymine transversion mutations, even though cancer promotion pathways remain unclear (Kapitulnik et al., 1978; Bartosiewicz et al., 2001; Baird et al., 2005; Hockley et al., 2009). BaP can also form hydroquinones that undergo a redox cycle generating genotoxic reactive oxygen species such as superoxide and hydrogen peroxide (Kim and Lee, 1997; Baird et al., 2005). Other potential mechanisms of toxicity and cancer promotion involve oxidative damage, immune suppression, activation of transposons, estrogen receptor activation, and disruption of cell-signaling (Romero et al., 1997; Vondrácek et al., 2002; Baird et al., 2005; Stribinskis and Ramos, 2006). Currently, the temporal ordering, specificity, and relative importance of these mechanisms in BaP toxicity is not fully elucidated.

In yeast, including *S. cerevisiae*, several cytochrome P-448 monooxygenase enzymes can perform Phase I metabolism on compounds like BaP and activate them to their toxic form (i.e., BPDE) in a manner analogous to mammalian microsomes (King et al., 1983; Käppeli, 1986). These yeast microsomes are indeed similar in weight and function to those observed in other mammalian species, including humans, but are enzymatically less efficient and the microsomes more rapidly lose activity when isolated *in vitro* (Azari and Wiseman, 1981; Käppeli, 1986). Moreover, the genetic expression of this rudimentary metabolism is not regulated by chemical exposure, but rather by a complex and unclear interaction between the presence of oxygen and the type of metabolism the yeast is undergoing at the time of growth (King et al., 1983). While it is clear that *S. cerevisiae* have the enzymatic capability to activate BaP into the toxic metabolites observed in other organisms including humans, the extent of activation has not been examined *in vivo* and the value of using a microsomal supernatant S-9 fraction solution as a metabolic adjuvant for bioactivation has not been assessed (Käppeli, 1986).

We used a functional genomic approach in yeast to identify the genes important for resistance or sensitivity to BaP exposure. Yeast is an useful model for functional assessment of the genetic requirements in toxicology. The yeast genome is fully characterized, primary biological functions are conserved and the relatively rapid reproductive rate of yeast enables exposures over multiple generations. In addition, deletion strains were generated through systematic insertion of “molecular barcodes” that individually identifies each gene knockout. As a result, functional genomic approaches in which entire sets of deletion strains can be pooled and simultaneously assayed for growth effects are possible (Giaever et al., 2002). In contrast to correlative genomic approaches such as transcriptomics, functional genomics directly identifies the genes tied to a phenotypic outcome (such as growth or toxicity). We have previously used this approach with several toxicants and shown the capability to translate results from yeast to mammalian systems (Jo et al., 2009a,b; Zhang et al., 2010; Ren et al., 2011). We also performed temporal protein expression profiling of yeast stress response. We used a selected set of yeast strains expressing full-length, chromosomally tagged green fluorescent protein fusion (GFP) proteins with genes encoding general stress response, oxidative stress response, chemical stress response, protein stress response, and DNA stress response (Huh et al., 2003). This approach allows measurement of real-time protein expression changes in response to stress. The selected stress response genes are present and highly conserved in most cell types of metazoans and are activated at significantly lower toxicant concentrations than those causing overt cellular injury (Kultz, 2005; Simmons et al., 2009).

## Materials and Methods

### Functional genomic analysis

#### Yeast strains

Diploid yeast deletion strains based on the BY4743 background (Invitrogen Corporation, Carlsbad, CA, USA) were used both for the parallel analysis pools (*n* = 4,757) and individual strain growth assays. For both the deletion strain pools and the individual strains, growth assays were performed in liquid media (2% peptone, 2% dextrose, and 1% dextrose by volume).

#### Yeast growth curves

In order to assay yeast response to BaP, yeast strains were grown to mid-log phase, diluted to an optical density of 0.0165 at 600 nm (OD_600_), and dispensed to a 48-well plate (non-treated polystyrene, Grenier Bio-One, Monroe, NC, USA). BaP (Sigma-Aldrich, St. Louis, MO, USA) was subsequently added to each well to the desired final concentration. At least two replicates for each dose and three biological replicates for each strain were performed. Each plate was subsequently incubated in a GENios microplate reader (Tecan, Durham, NC, USA) set to 30°C with intermittent shaking and OD_595_ measurements at 595 nm were recorded every 15 min for 24 h. The raw absorbance data obtained was corrected for background, averaged across replicates, and plotted against time. Excel 2010 (Microsoft Corporation, Redmond, WA, USA) was used to calculate the area under the curve (AUC) as a measure of growth (the outcome of interest) and to perform statistical analyses, namely, comparing AUC values by means of Student’s *t*-test, assuming constant variance between samples.

#### Benzo[a]pyrene stock solutions

BaP was solubilized in dimethylsulfoxide (DMSO) and the final concentration of DMSO in the yeast media never exceeded one percent by volume, the standard DMSO concentration at which no observable effect occurred on yeast. Backgrounds and controls included one percent DMSO to account for any potential effect. The BaP stock solutions were stored in the chemical hood shielded from light. As long as it is shielded from light, BaP is stable in DMSO solutions, with less than 20% degradation over 3 months at 25°C (Dabrowska et al., 2008).

#### IC_20_ determination

Initially, using the method developed by Pierce et al. (2007), the concentration of BaP which resulted in a 20% decrease in growth in wild type yeast (IC_20_) was determined. The IC_20_ point was chosen for its ability to affect yeast deletion strains measurably and differentially without incurring the loss of toxicant specificity that occurs at higher concentrations (Pierce et al., 2007).

#### Yeast parallel deletion analysis

Targeted inactivation of a gene, “knockout,” is often used to investigate the gene’s function. Similarly, this same technique can be used to identify how a gene or lack thereof affects survival of a model organism in response to a toxicant (Giaever et al., 2002). Using a gene knockout library of *S. cerevisiae* strains that represent 96% of the yeast genome’s open reading frames (ORF), we can thus determine in parallel the effect of each gene on the growth outcome as it relates to BAP exposure (Giaever et al., 2002). This is accomplished by way of unique DNA “molecular barcodes” that correspond to each knockout strain. After varying time and dose exposures to BAP, the pooled yeast knockout library cells can then be harvested and the extracted DNA barcodes hybridized to oligonucleotide arrays, thereby allowing for a quantitative determination and comparison of growth outcome for each individual strain/gene (Giaever et al., 2002).

To this end, after determining the IC_20_, pool growth, genomic DNA extraction, and array hybridization were conducted as described by Pierce et al. (2007), with a few minor changes described herein. In summary, viable homozygous diploid deletion mutant strains of yeast (*n* = 4,757) were continuously subjected to three different BaP concentrations (IC_20_, 50% of the IC_20_, and 25% of the IC_20_) over a growth period of 5 and 15 generations (5G and 15G). In this way, three total biological replicates were obtained for each dose and generation time period. After collecting the cells, the genomic DNA was extracted with the YDER kit (Pierce Biotechnology, Rockford, IL, USA). The unique barcodes in the DNA, specific to each yeast strain, were amplified by PCR using a set of biotinylated primers, and the resulting products were then hybridized to TAG4 arrays (Affymetrix, Santa Clara, CA, USA).

#### Differential strain sensitivity analysis

In order to investigate genes required for optimal growth of yeast in the presence of BaP, differential strain sensitivity analysis (DSSA) was used to identify differential growth of viable yeast knockout strains relative to wild type yeast in the presence of the toxicant. To this end, the data obtained from the TAG4 array was log_2_ transformed, adjusted for signal saturation as described in Pierce et al. (2007) as well as adjusted for mean chip background by means of robust location and scale estimators for log_2_ transformed amplitudes of null features (18,000 total, evenly spread on the array). To control for variability in strain growth, results from each treatment array were matched to data from 12 controls (5G or 15G) for analysis. Treatment-control pairs were normalized with locally weighted scatterplot smoothing (global normalization), and the difference in growth between strains was identified using an alpha-outlier approach (Loguinov et al., 2004). Data from three biological replicates were combined, resulting in 36 treatment-control data pairs per treatment group. Residual variances (with a robust/* scale estimator) of log_2_ (treatment/control) for each 36 pairs were inspected using box plots. The “effective pairs” were then determined by excluding pairs with abnormally high residual variance, or with suspected serial correlation in variability (regular patterns in the box plots).

Significant yeast deletion strains (i.e., genes) were statistically inferred by an exact binomial test, with the assumption that the outcomes for each gene in all treatment-control pairs were independent binary variables with the same probability of success for all Bernoulli trials. For a particular gene n, outcomes were considered as “successful” if they were significant in the outlier analysis with *q*-values ≤0.05 in each of all effective pairs with log_2_ ratios of the same sign, simultaneously (Loguinov et al., 2004). The corresponding raw *p* values based on the exact binomial test were then corrected for multiplicity of comparisons using a *q*-value approach and only the genes with *q*-value ≤0.05 were considered for further analysis. This approach does not apply a scale estimator and, as a result, it did not require between-chip pair normalization for the statistical inference. Subsequently, a fitness score (ave[log_2_{Y|X = exposed}] – ave[log_2_{Y|X = control}]), the difference of the means of the (log_2_) hybridization signal between exposed and control was determined for each yeast deletion strain. Negative scores indicate sensitivity of the strain to BaP exposure; conversely, a positive score indicates resistance to BaP exposure. Consequently, in the case of a negative score, the gene product deleted from that strain is likely required for resistance to the toxicant. Similarly, in the case of a positive score, the absent gene product may serve to sensitize the cell to BaP (North et al., 2011).

#### Gene ontology

From the significant genes responsible for resistance or sensitivity to BaP in yeast identified in the DSSA method described above, overrepresented Gene Ontology^1^ (GO) and MIPS (Munich Information Center for Protein Sequences^2^) categories were identified to determine which specific (if any) gene clusters related to biological characteristics were relevant to *S. cerevisiae*’s response to BaP exposure (Ashburner et al., 2000; Mewes et al., 2002). To this end, the Functional Specification resource, FunSpec^3^, was used in an enrichment analysis to determine the significantly represented GO and MIPS biological categories among the sensitive/resistant strains (Robinson et al., 2002). We used the hypergeometric distribution of FunSpec with a *p*-value significance threshold of 0.01.

#### Selected confirmation of yeast strain significance

In order to confirm the data previously obtained, and based on the results from the DSSA and GO, select relevant significant yeast gene deletion strains were chosen for individual testing. Yeast growth curves, as described above, were performed at two different BaP concentrations (100% IC_20_, 25% IC_20_) for the chosen deletion strains.

#### S-9 metabolic adjuvant

S-9 (Molecular Toxicology, Inc., Boone, NC, USA) was obtained as 30% by volume S-9 rat liver microsome mix (NADP, d-glucose-6-phosphate, MgCl2, and KCl co-factors; pH = 7.4 sodium phosphate buffer) containing all co-factors for a sustained enzymatic *in vitro* metabolic activation. The S-9 was reconstituted with nuclease-free water and stored at −20°C. The S-9 was prepared and mixed with the liquid YPD media immediately prior to dispensing into each of the 48-well plates. The final concentration in each well was either 1 or 2% by volume, based on a prior study which suggested 1.85% by volume as the optimal S-9 concentration for the *in vitro* activation of BaP with S-9 (Hakura et al., 2001). The yeast growth curve protocol was followed as described previously and the results controlled for background absorption and/or toxicity due to S-9. Our statistical analysis of the results included the use of two-way ANOVA to assess BAP by S-9 interaction as well as the relevant Tukey *post hoc* tests.

### Proteomic analysis of stress response pathways

#### GFP-fused yeast strains and stress response ensemble

We employed a library of in frame GFP fusion proteins (Invitrogen, no. 95702) of *S. cerevisiae* (ATCC 201388), constructed by oligonucleotide-directed homologous recombination to tag each ORF with *Aequrea victoria* GFP (S65T) in its chromosomal location at the 3′ end. A set of stress response pathway genes, with a total of selected 123 ORFs in yeast, was selected to cover five different functional categories of stress response (Table 1).

**Table 1:** **Cellular stress response pathways ensemble of ORFs for toxicity response in GFP-tagged yeast**.

Stress	Function	Pathway	Protein involved
General stress response	Metabolism	Trehalose synthesis	TPS1, TPS2, TSL1, NTH1
		Osmotic stress	HOG1, GPD1, SLN1, MSB2
		Other metabolism	GSY2, HXK1, GLK1, PFK26, FBP26
	General function	Signal transduction	PGM2, TPK1, TPK2, CDC28
		apoptosis	YAP1, CDC48, CDC6, MCA1, NMA111, Tat-D, FIS1, OYE2, YSP2
	Electron and energy related		COX17, COR1, CYC7, GND2
	Other response (hypoxia, etc)		CYC7
Oxidative stress	Redox	Sensor/regulon	YAP1, SLN1, SKN7, MSN2
		SOD	SOD1
		Catalase	CTT1
		Glutaredoxin	GRX1, GRX2
		Glutathione	GSH1, GPX1, GTT1, HYR1, ECM38
		Thioredoxin	TRX2, TSA2
		Others	ISM1, ATM1, CCP1, PRX1
Chemical stress	Membrane, cell wall and cell structure		PUN1, HSP12, MSN2
	ABC transporters or related		PDR1, PDR3, YCF1, PDR5, SNQ2, BPT1, ATM1
	MFS transporter (H + antiporters)		FLR1, QDR2, ATR1
			TPO1, AQR1, QDR3, TOP2
	Antibiotic resistance		YRR1, YRM1
	Other detoxification	Metal	BSD2
		Steroid	ATF2
		NO	YHB1
		Others	ECM38
Protein stress	Protein misfolding	Heat shock related protein	HSP104, HSP42, HSP78, HSP26, SSA4, SSA3, SSE2
		Cold shock	TIR1, TIP1, BFR2
	ER stress	Sensor; transcription factor	IRE1; HAC1
	Degradation and vacuolar function	Ubiquitin	UBC5, UBC8, HSP26
		Autophagy	ATG1, UTH1
DNA stress	DNA repair	Damage signaling	CHK1, MMS2
		Direct repair	PHR1
		BER	OGG1, NTG1, NTG2, UNG1, MAG1
			RAD27, APN1, APN2
		NER	RAD1, RAD2, RAD4, RAD9, NAD14, RAD16, RAD23, RAD30, RAD34
		MMR	MSH1, MSH2, MSH3, MSH6, PMS1, MLH1, MLH2
		DSB: general	XRS2, MRE11
		HR	RFA1, RFA2, RFA3, RAD51, RAD52, RAD54, HTA1, HTA2
		NHEJ	LIF1, YKU70

#### BaP solution

BaP was solubilized in dimethylsulfoxide (DMSO) as described in Section “Benzo[a]Pyrene Stock Solutions,” with rat liver extraction S-9 (Invitrogen, Grand Island) added to obtain a final concentration of 1.4% for bioactivation before exposure as previously discussed. Sub-cytotoxic doses from 0.01 to 1000 mg/L (below IC_5_ = 3198.89 mg/L, with >95% survival percentage for 24 h exposure by AUC method in Section “Yeast Growth Curves”) were applied.

#### Real-time transcriptional analysis

A total of six dose concentrations across 6-logs below IC_5_ were tested using the GFP-tagged library and shown in a heat map. To allow for comparison, we focused our discussion mainly with two concentrations, 10 and 100 mg/L, since these two concentrations are close to those used (83.26 mg/L, 20.82 mg/L) in the deletion library investigation. This study aims to reveal the toxicity mechanism of BAP, so dose concentrations below IC_5_ but sufficiently high enough to lead to observable molecular disturbance were chosen. We note that there is bio-accumulation of BAP in organisms of four orders of magnitude on average, so cells are likely exposed to much higher concentrations than those typically found in the environment (Janikowska and Wardas, 2002; Wang and Wang, 2006).

GFP tagged yeast strains selected were grown in clear bottom black 384-well plates (Costar) with SD medium at 30°C until the cultures reached early exponential growth (OD_600_ about 0.2–0.4). Ten microliters of BaP-S-9 mixture was added per well to obtain the final concentration from 1000 to 0.01 mg/L with 10× dilution. PBS-S-9 mixture of equal volume was used as vehicle control. Plates were put in a Micro plate Reader (Synergy H1 Hybrid Multi-Mode, Biotek, Winooski, VT, USA) to read continuously for absorbance (OD_600_ for cell growth) and GFP signal (filters 485 nm, 528 nm for protein expression) every 5 min for 2 h (with orbital shake at high speed for 1 min before each reading). All tests were performed in triplicate.

#### Data analysis

OD and GFP raw data from measurement were corrected by background OD and GFP signal of medium control (with or without chemicals). The protein expression for each measurement is then normalized by cell number (OD_corrected_) as *P* = (GFP_corrected_/OD_corrected_). The *P* level was corrected with internal control for plate normalization (details not included here). Then the alteration in expression for a given protein at each time point due to chemical exposure, also referred as induction factor I, was represented as *I* = *P*_experiment_/*P*_vehicle_ by corrected values.

To quantify the chemical-induced protein expression level changes of a treatment, the real-time protein expression profile was then integrated into the Protein Expression Level Index (PELI) as following:

For protein i, the accumulative protein expression over their 2 h exposure period, which is directly related to its function, is calculated as

$$\text{PEL}{\text{I}}_{\text{ORFi}}=\frac{\int_{t=0}^{t}Idt}{\text{exposure}\;\text{time}},$$

An assay noise cut-off is determined using a signal-to-noise ratio of 1.5. For triplicates PELI_ORF_ were evaluated by Mean ± SD.

#### Gene ontology

Gene ontology analysis was performed with novel Network Ontology Analysis method (NOA) for enrichment analysis to determine the significantly represented GO biological categories, and to analyze functions of gene network, as it allows enrichment analysis with user defined reference set (Wang et al., 2011). In this study the whole stress library was used as the reference set, and activated ORFs were used as test set, which is defined as PELI_ORF_ > 1.5 based on signal-to-noise ratio of the test. A *p*-value significance threshold of 0.01 was used similar to the deletion library.

## Results

### BaP IC_20_ determination

**Figure 1:**
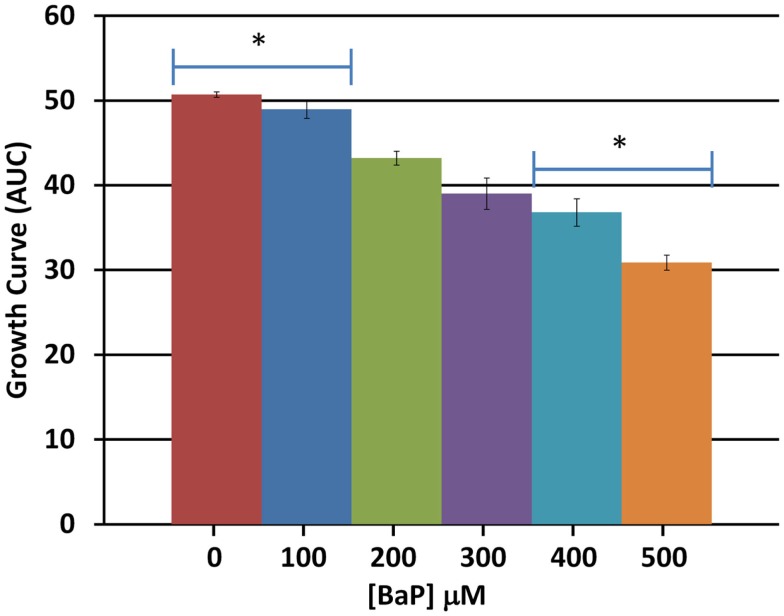
**Average total cell growth**. Average total cell growth over the 24 h period was obtained by calculating the area under the curve (AUC) for each dose and averaging the result across three biological replicates. Bars are standard errors of three biological replicates; significance by *t*-test comparison between each two doses sequentially; *represents *p* < 0.05.

**Figure A1:**
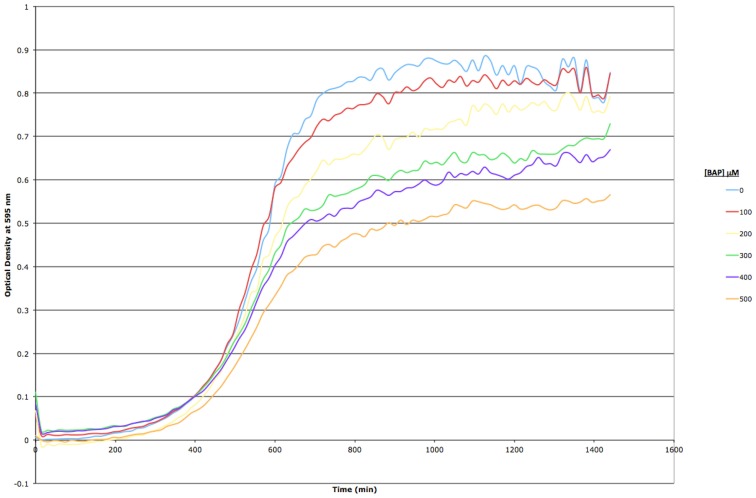
**Growth curve assay for BY4743 wild type *S. cerevisiae* exposed to increasing concentrations of BAP in YPD liquid media**. Optical density at 595 nm was measured every 15 min for a period of 24 h; each point in the curve represents a mean of three replicates. Standard error is omitted for clarity.

**Figure A2:**
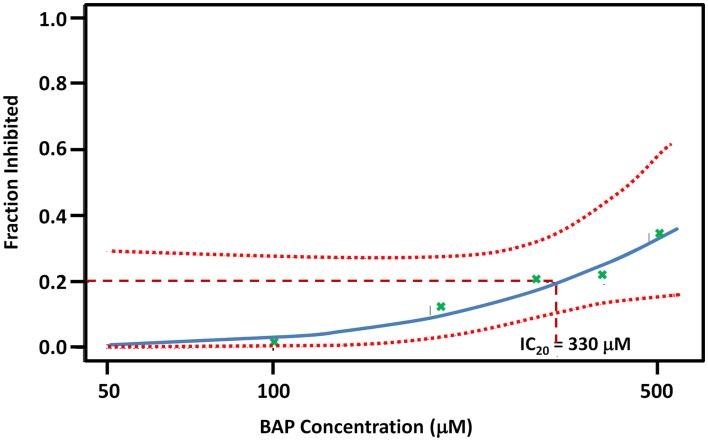
**BAP Dose-response curve**. Each “bp” green x mark is a point estimate of three biological replicate exposures at five concentrations: 100, 200, 300, 400, and 500 μm. The dotted line represents 95% confidence interval to the fitted line. See Figure A1 for growth curves and total growth data for each of the five doses.

Based on a literature search of available toxicity data and empirical determination, we used the yeast growth curve assay to analyze the growth response of wild type BY4743 yeast in liquid YPD media as a function of BaP concentration by measuring OD_595_ every 15 min for 24 h. Total cell growth at each chemical dose was then determined by calculating the AUC of each OD versus time growth curve plot (Figure A1 in Appendix; Figure 1). Visual inspection of Figure A1 in Appendix reveals that most of the BaP toxicity dose-response is expressed later during the stationary growth phase, following exponential growth rather than from initial exposure. Moreover, from this assay, the BaP IC_20_, or exposure concentration resulting in a 20% inhibition of growth relative to the absence of chemical treatment, was determined to be 330 μM (Figure A2 in Appendix). From the IC_20_ value thus calculated, we defined two other concentrations to be used subsequently in DSSA as exposure concentrations for the pooled homozygous diploid yeast deletion mutants exposure periods of five and 15 yeast generations (5G and 15G; Table 2).

**Table 2:** **Concentrations used in DSSA pooled homozygous diploid yeast deletion mutant exposures to BaP**.

Name	BaP dose concentration (μM)
IC_20_	330
50% IC_20_	165
25% IC_20_	82.5

### Differential strain sensitivity analysis

**Figure 2:**
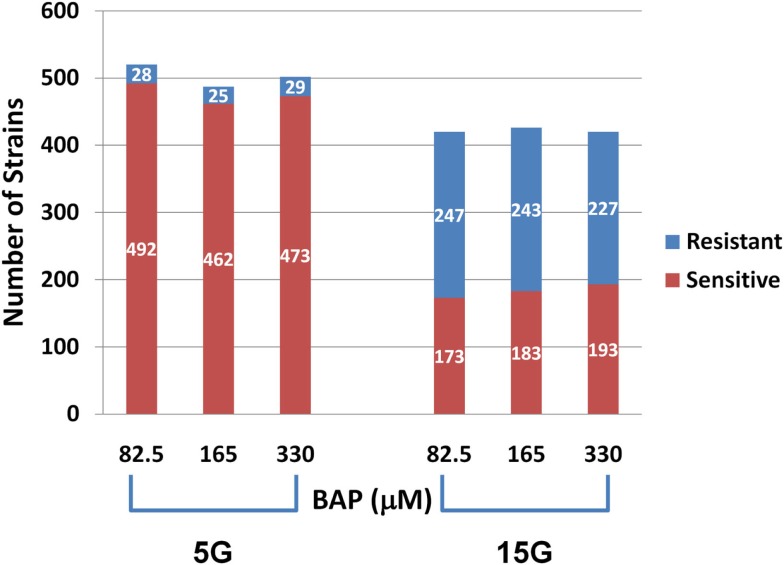
**Number of sensitive and resistant yeast deletion strains for each exposure at three BaP concentrations and for five (5G) and 15 (15G) generations**.

Differential strain sensitivity analysis, described in the methods, was performed at the concentrations specified in Table 1 for 5G and 15G exposure periods to identify genes, each represented by a yeast deletion strain, required for resistance or sensitivity to BaP exposure. The results from this assay indicate that the number of yeast genes conferring resistance or sensitivity did not vary by dose for a 5G or 15G exposure, which may be indicative of a sharp threshold response (Figure 2). We note approximately 17-fold more strains exhibiting BaP sensitivity relative to BaP resistance in the 5G exposure (Figure 2) while in the 15G exposure there are more resistant strains than sensitive ones (Figure 2). Overall, more strains responded to BaP exposure during a 5G than during a 15G exposure (Figure 2).

**Figure 3:**
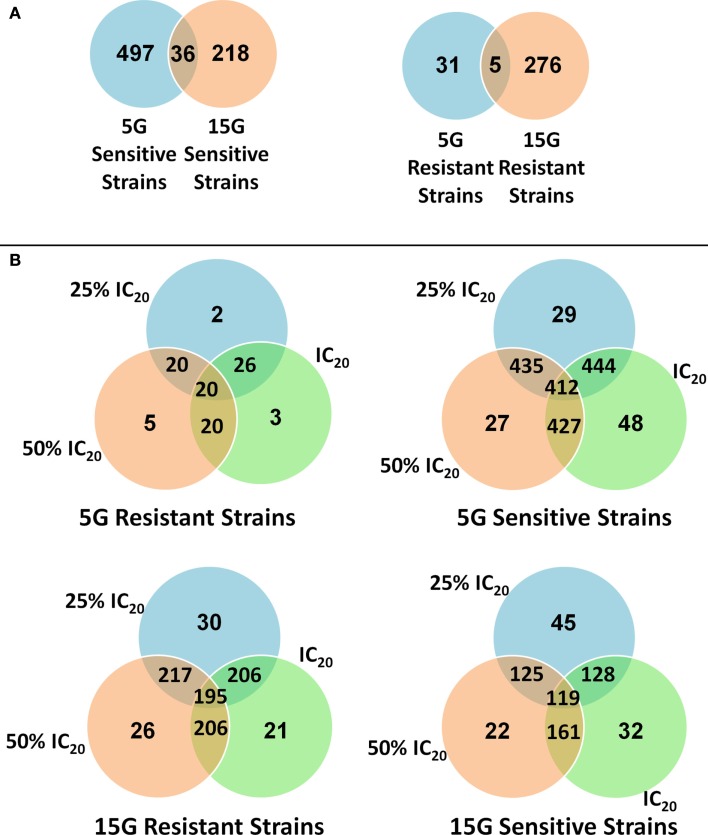
**Venn diagrams of the amount and overlap of sensitive/resistant strains observed across overall exposure duration categories (A) and for each dose group (B)**.

We found many strains commonly sensitive or resistant in each dose exposure group (5G or 15G) but there was a much smaller overlap between the 5G and 15G exposures (Figure 3).

Overall, 310 yeast mutant deletion strains were resistant and 753 were sensitive to BaP under at least one of the six treatment conditions (three doses, two time periods). Of these sensitive and resistant strains, 209 were resistant and 528 were sensitive under at least three treatment conditions (Tables S1–S4 in Supplementary Material). The treatment results for the most significant deletion strains are reported in Tables 3 and 4.

**Table 3:** **Table of the 25 most sensitive yeast strains exhibiting significantly negative log_2_ values during a 5G exposure treatment**.

		Treatment		
		5G		
		82.5 μM	165 μM	330 μM

Yeast gene name	Open reading frame/Yeast deletion strain	25% IC_20_	50% IC_20_	100% IC_20_
*GAT1*	YFL021W	−5.6	−5.5	−5.7
*GRX6*	YDL010W	−4.7	−4.6	−4.7
*RPL33B*	YOR234C	−4.6	−4.6	−4.6
	YBR138C	−4.5	−4.6	−4.6
	YFL019C	−4.6	−4.4	−4.6
	YBL065W	−4.4	−4.1	−4.4
*PGM2*	YMR105C	−4.2	−4.3	−4.2
*ECM3*	YOR092W	−4.2	−4.1	−4.3
*MRPL39*	YML009C	−4.3	−4.1	−4.2
	YML079W	−4	−4.1	−4.1
	YNL217W	−4	−4.1	−3.8
*DCS2*	YOR173W	−3.9	−3.9	−4
*MRP2*	YPR166C	−3.9	−4	−3.9
*TPO5*	YKL174C	−4	−3.8	−3.9
*HRQ1*	YDR291W	−3.9	−3.8	−3.8
*SLM2*	YNL047C	−3.8	−3.8	−3.9
	YML119W	−3.8	−3.8	−3.8
*MAP1*	YLR244C	−3.8	−3.9	−3.7
	YGL114W	−3.7	−3.6	−3.7
*SSF2*	YDR312W	−3.6	−3.7	−3.7
	YJR079W	−3.8	−3.5	−3.6
	YJL046W	−3.6	−3.7	−3.6
	YOL118C	−3.6	−3.6	−3.6
*FMT1*	YBL013W	−3.6	−3.5	−3.6
	YMR119W−A	−3.4	−3.5	−3.5

*Larger negative values indicate greater sensitivity to BaP*.

**Table 4:** **Table of the 25 most sensitive yeast strains exhibiting significantly negative log_2_ values during a 15G exposure treatment**.

		Treatment		
		15G		
		82.5 μM	165 μM	330 μM

Yeast gene name	Open reading frame/Yeast deletion strain	25% IC_20_	50% IC_20_	100% IC_20_
*BNA5*	YLR231C	−4.4	−4.4	−4.8
*DMA1*	YHR115C	−4.2	−4.6	−4.7
*AST2*	YER101C	−4.3	−4.4	−4.5
	YML079W	−4.3	−4.2	−4.6
*RMD5*	YDR255C	−4.2	−4.2	−4.3
*ACE2*	YLR131C	−3.3	−3.35	−5.6
*NMA2*	YGR010W	−3.5	−4.1	−4.1
*RPS4A*	YJR145C	−3.7	−3.8	−4
*PET309*	YLR067C	−3.7	−3.8	−4
*LEM3*	YNL323W	−3.4	−3.7	−3.6
*ECM32*	YER176W	−3.2	−3.7	−3.7
*SRO9*	YCL037C	−3.3	−3.6	−3.6
	YGL041C	−3.2	−3.3	−3.8
*TCB1*	YOR086C	−3.3	−3.4	−3.4
*BUG1*	YDL099W	−3.1	−3.4	−3.6
*AHP1*	YLR109W	−2.9	−3.5	−3.6
	YNL285W	−2.9	−3.1	−3.3
	YMR158W-A	−2.8	−3.2	−3.2
*SGA1*	YIL099W	−2.8	−3	−3
*SIS2*	YKR072C	−2.8	−3	−3
*TGL4*	YKR089C	−2.9	−2.8	−3.1
*GSP2*	YOR185C	−2.6	−3	−3
*ASG1*	YIL130W	−2.9	−2.8	−2.8
*RPI1*	YIL119C	−2.5	−3	−2.9
*RNH203*	YLR154C	−2.4	−3	−3

*Larger negative values indicate greater sensitivity to BaP*.

### Gene ontology

From the strains identified in Tables S1 and S2 in Supplementary Material, an enrichment analysis was performed to identify significantly overrepresented categories of genes among the sensitive/resistant strains. The overrepresented categories are summarized in Table S5 in Supplementary Material. Based on the results of Table S5 in Supplementary Material, the significance of the categories represented, and a review of BaP toxicity, we identified select strains associated with biological categories of potential relevance to BaP toxicity. The strains of interest are reported in Table 5, a subset of Table S5 in Supplementary Material. Genes related to transmembrane transport, mitigating reactive oxygen species, and DNA repair were identified as of particular relevance to BaP toxicity.

**Table 5:** **Overrepresented (*p*-value < 0.01) biological categories based on the MIPS and GO databases of the Functional Specification Instrument for 5G and 15G exposures of select significantly resistant and sensitive strains, as well as any observed overlap strains between 5G and 15G (Ashburner et al., 2000; Mewes et al., 2002; Robinson et al., 2002)**.

Category	*p*-value	Name of genes in category	Number of genes observed in category	Number of genes in category
**5G SENSITIVE**				
**GO Molecular Function**
Transferase activity [GO:0016740]	0.000779	*KIN3 FMT1 ALG3 CST26 EHT1 DPB3 KCC4 FEN1 BUD23 MGT1 KIN1 MTQ2 AKR1 TRP4 DOT1 EMI2 HIS1 TMT1 RIM15 CMK1 YFR018C TAN1 NAT2 CHO2 STE20 ARD1 YCK1 PFK26 HPM1 POT1 DAL7 AIM22 IKS1 TPK1 SET2 YJL218W ELM1 AAT1 TPK3 KTR2 AYT1 **SHM2** RCK2 NNT1 TAL1 SUR4 ERG6 TRM9 APT1 URA5 TDA1 CPT1 ARE2 PFA4 GAS5 PKH2 PAP2 OST3 ABP140 TUM1 MEK1 SKS1 BTS1 ISR1 TAZ1 GPH1*	66	611
NADPH dehydrogenase activity [GO:0003959]	0.005429	*OYE2 **OYE3***	2	2

**GO Biological Process**				
Chromatin silencing at telomere [GO:0006348]	0.002979	*SWD1 DPB3 DOT1 DOT6 **RAD6** ASF1 SPT10 IES3 MEC3 YAF9 SAS5*	11	58

**MIPS Functional Classification**

Meiotic recombination [10.01.05.03.01]	0.005619	*MSH4 DST1 SAE2 RIM4 SAE3 REC104 RAD52 **RAD50***	8	38
Organization of chromosome structure [42.10.03]	0.0058	*SWD1 RIF1 SGF29 SPT3 DOT1 IES1 EST3 SET2 NAP1 IES3 SPT8 RAD52 YKU70 **RAD50***	14	90
Cytoplasm [725]	0.001171	*ATS1 DRS2 CLN3 FLC2 APN2 PIN4 ALG3 SRO77 UGA2 GAL10 SCO2 RPS11B UBP14 TAT1 SLM4 RPL19A OPY1 YBR138C ARA1 ARL1 PEX32 EHT1 YBR225W UBX7 DPB3 DCC1 FRM2 FEN1 BUD23 RPN4 YDL063C PEX19 BDF2 RPL13A CYK3 RDI1 PPH22 RTN2 YDL211C GDH2 **DTD1** FMP45 PTP1 YDL241W GRX3 KIN1 MTQ2 RSM24 COQ4 BTT1 AKR1 HNT2 ASP1 YDR336W YDR338C YPS7 TRP4 EFT2 SAC7 URH1 SIZ1 CYM1 DOT1 SSN2 YDR444W RPS18A EMI2 IRC4 GIM4 GLY1 YEL047C TCA17 DOT6 SWI4 SLX8 YER137C RTR1 YER156C YER163C TMT1 DEG1 VTC2 BLM10 WWM1 YFL012W HSP12 LPD1 GAT1 BUD27 RIM15 RPL22B FET5 EMP47 CMK1 YFR016C LSB3 DUG1 **RAD6** RPL9A SAE2 TAN1 SCM4 YGR117C NAT2 RPL24B CHO2 YHB1 GND2 RAD2 SLH1 STE20 RIM4 SNF6 ARD1 YHI9 AAP1 YCK1 OYE2 GND1 RPN10 YIL089W PFK26 HPM1 HOS4 POT1 MET28 DAL81 YVH1 HYR1 MAD3 YJL043W TDH1 YJL055W IKS1 RPE1 SPT10 RPS21B TPK1 REE1 YJL218W CPR7 YMR1 YJR111C YJR154W YKL070W KTI12 PMU1 RPS27A TPK3 SAC1 YKR041W NAP1 MSA2 YKR078W MLP1 YKR096W TPO1 MLH2 YLR036C **SHM2** CCW12 ACE2 TFS1 HCR1 CCC1 MAP1 RCK2 NNT1 RPS30A TMA10 VRP1 NIT3 TAL1 ATG33 DIF1 RPL6B YLR456W ERG6 TRM9 APT1 YOX1 YML079W URA5 YMR074C PGM2 YMR114C ATG16 YMR244C-A GFD1 YKU70 TDA1 YMR295C DYN3 ELP6 PUB1 SLM2 MLF3 OCA1 YAF9 YNL108C YNL122C JJJ1 **RAD50** PDR17 CAF120 HCH1 PUS4 KRE1 SIN3 PKH2 RPS19A WHI2 VHS3 DCS2 SAS5 RPL33B ABP140 TUM1 HNT3 CAF20 RPL20B VTS1 LSP1 CHL1 TAE2 HST2 SKS1 SVL3 SRL4 ARL3 BTS1 RPL21B SSE1 GDE1 BEM4 **OYE3** FLC1 NEW1 KEL3 ANT1 NCE102 GPH1 MET16 YPR174C AQY1*	245	2879
**15G SENSITIVE**				
**GO Molecular function**
Glycine hydroxymethyltransferase activity [GO:0004372]	0.001125	*SHM1 **SHM2***	2	2
Pyridoxal phosphate binding [GO:0030170]	0.002904	*SHM1 ALT2 GLY1 IRC7 **SHM2** BNA5*	6	43
Zinc ion binding [GO:0008270]	0.004272	*HIS4 ADH7 NRP1 NRG1 SAN1 AST2 GAT1 DMA1 AIR1 ASG1 GAT4 DAL81 ACE2 ECM22 CAT8 FAP1 GIS2 PFA4 **YRM1** ULS1*	20	314
Sequence-specific DNA binding transcription factor activity [GO:0003700]	0.00656	*GAT1 HAC1 XBP1 ASG1 GAT4 DAL81 PHD1 ECM22 CAT8 FAP1 **YRM1***	11	138
Metal ion binding [GO:0046872]	0.009408	*HIS4 ADH7 NRP1 NRG1 SAN1 GAT1 DMA1 HOP1 AIR1 ASG1 GAT4 DAL81 ELM1 ELF1 JLP1 IZH3 PDC1 EMP46 ACE2 ECM22 IRC21 CAT8 FAP1 NRK1 GIS2 FRE4 PFA4 **DNL4** TCB1 **YRM1** ULS1 FMP30 PDH1*	33	647
Sequence-specific DNA binding [GO:0043565]	0.009413	*NRG1 GAT1 HAC1 XBP1 ASG1 GAT4 PHD1 BAS1 ACE2 ECM22 CAT8 **YRM1***	12	165
Catalytic activity [GO:0003824]	0.009792	*SHM1 HIS4 ADH7 PHO13 TRP1 ARO3 TPS2 ALT2 GLY1 AST2 IRC7 SGA1 IRC24 LAS21 YKL071W YKL107W SIS2 PDC1 **SHM2** BNA5 EXG1 CDA1 GRE2 LSC1 GDB1*	25	455

**GO Biological Process**
l-serine metabolic process [GO:0006563]	0.001125	*SHM1 **SHM2***	2	2
Protein ubiquitination involved in ubiquitin-dependent protein catabolic process [GO:0042787]	0.003238	*SAN1 RMD5 UBC8 **ELC1***	4	19
Cell redox homeostasis [GO:0045454]	0.003414	***PRX1 TRX2 POR2 AHP1 GLR1***	5	31
Drug transmembrane transport [GO:0006855]	0.005065	*YDR338C **QDR2 YRM1***	3	11
Glycine metabolic process [GO:0006544]	0.006456	*SHM1 SHM2*	2	4

**GO Cellular Component**
Membrane [GO:0016020]	0.003977	*UIP3 SCS22 FIG2 MSS2 RGT2 RTN2 UGA4 ENT5 SPR28 OMS1 YDR338C ATP17 QCR7 YEA4 PIC2 YFR012W QCR6 KEX1 YPT32 **TRX2** PHB2 APL6 PUT2 HXT1 CBR1 FIS1 KTR7 YIL089W SLM1 **POR2 QDR2** ATG32 DAL4 LAS21 NUP100 YKL107W PXA2 CAF4 GAP1 TGL4 SPO75 YEH2 IZH3 PET309 EMP46 EMP70 ENT2 ECM22 YLR283W NUP2 SMA2 PLB1 YET2 ATP25 AIM36 INP2 YMR166C MFA2 LEM3 ATO2 FRE4 PFA4 TIR4 VPS5 TCB1 KTR1 FMP30 COX11 ATG5 UIP4 FLC1 SAM3 JID1 YPR174C*	74	1671

**MIPS Functional Classification**
C-1 compound anabolism [01.05.05.04]	0.003301	*SHM1 SHM2*	2	3
Detoxification by export [32.07.05]	0.003301	***QDR2 YRM1***	2	3
Oxygen and radical detoxification [32.07.07]	0.006587	***PRX1 TRX2 AHP1***	3	12

**MIPS Subcellular Localization**
Cytoplasm [725]	0.002267	*UIP3 **PRX1** SEA4 NGR1 FRM2 HIS4 SRO9 YCL042W ADH7 BUG1 MSS2 UBP1 LDB17 NRP1 YDL177C RTN2 **DTD1** PHO13 TRP1 ARO3 NRG1 TPS2 BMH2 ALT2 GIR2 ENT5 CSN9 RMD5 YDR336W YDR338C SSN2 RMT2 UBC8 YEL043W GLY1 SAP1 AST2 ECM32 GAT1 HAC1 FAR7 QCR6 KEX1 YPT32 NMA2 ASN2 YGR153W **TRX2** APL6 EFM1 ARD1 DMA1 LSM12 TDA11 AIR1 YIL089W XBP1 SLM1 RPI1 DJP1 DAL81 IRC24 RPS4A YKL063C YKL071W YKL091C YKL107W TPK3 NAP1 TRM2 SIS2 TGL4 YLR031W PDC1 **SHM2 AHP1** ACE2 RNH203 YKE2 CPR6 ECM22 BNA5 RPN13 DAK1 YML079W PLB1 IRC21 RPL15B YMR124W YMR147W ATG16 YMR166C ZDS1 CAT8 PUB1 NRK1 NPR1 GIS2 LEM3 SSK2 PKH2 GRE2 SHE4 CKA2 VPS5 EFT1 **YRM1** GSP2 PAC1 TAE2 **ELC1 GLR1** RTT10 UIP4 FLC1 PDH1 YPR174C GDB1*	118	2879

**MIPS Protein Complexes**
Complex Number 160 [550.2.160]	0.000146	***AHP1** MEC3 **DNL4***	3	4
**15G AND 5G SENSITIVE OVERLAP**				
**GO Molecular Function**
d-tyrosyl-tRNA(Tyr) deacylase activity [GO:0051500]	0.004998	***DTD1***	1	1
Glycine hydroxymethyltransferase activity [GO:0004372]	0.009971	***SHM2***	1	2

**GO Biological Process**
d-amino acid catabolic process [GO:0019478]	0.004998	***DTD1***	1	1
l-serine metabolic process [GO:0006563]	0.009971	***SHM2***	1	2

**MIPS Protein Complexes**
Complex Number 2, probably cell cycle [550.1.2]	0.009971	***DTD1***	1	2

*All strains included in this analysis were sensitive in at least one of the six treatments. Bolded genes correspond to those whose response to BaP was confirmed by individual exposure of the corresponding deletion strain*.

### Validation of DSSA data

**Figure A3:**
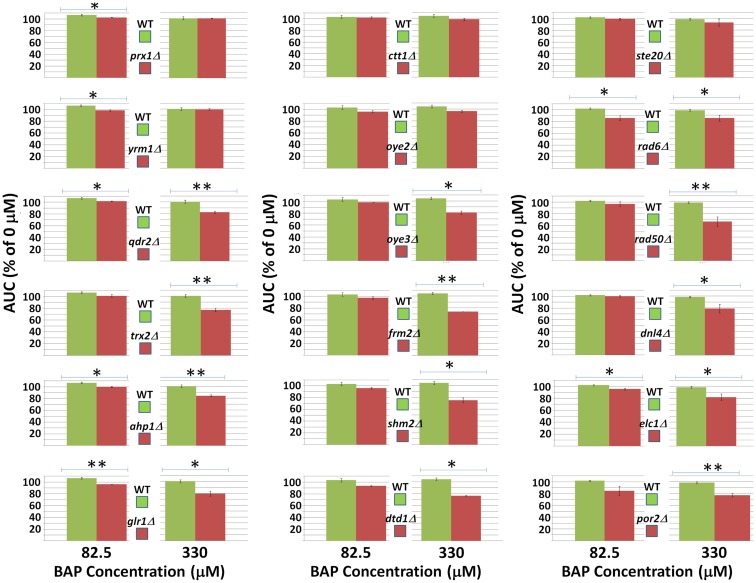
**Confirmation of inhibition of selected yeast strains exposed individually to BAP**. *Represents significance at 0.01<p<0.05; **represents significance at p<0.01. Bars are standard errors.

From the DSSA results previously described, 18 yeast deletion strains that exhibited significant resistance/sensitivity to BaP exposure were chosen to validate results using individual assays. These 18 deletion strains were independently exposed to BaP concentrations of 0% IC_20_ (0 μM), 25% IC_20_ (82.5 μM), and 100% IC_20_ (330 μM) for 24 h, following the same protocol as the yeast growth curve assay used to identify the IC_20_. Subsequently, growth inhibition at the various concentrations was calculated in relation to the AUC of each growth curve, in an analogous manner as the IC_20_ determination. Results and statistical significance of the validation analysis are included in Figure A3 in Appendix. All of the strains tested demonstrated statistically significant inhibition using at least one BaP concentration relative to the 0% IC_20_ control, with the exception of *ste20*Δ, *ctt1*Δ, and *oye2*Δ whose growth inhibition was not statistically significant. Moreover, of the fifteen strains that demonstrated statistically significant inhibition, only *glr1*Δ, *prx1*Δ, and *yrm1*Δ did not show a dose-dependent growth inhibition response with inhibition increasing concomitantly with dose. The strains whose sensitivity to BaP was confirmed in this assay are the same as the bolded strain subset listed in Table 5.

### S-9 metabolic activation

**Figure 4:**
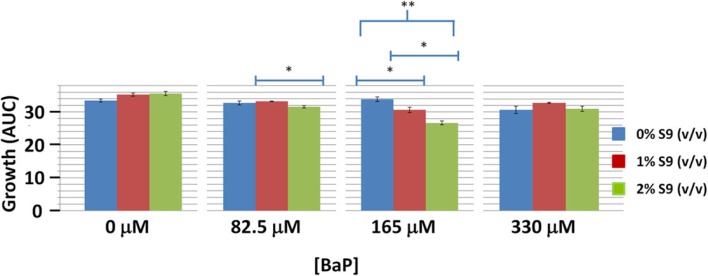
**Wild type yeast exposure to various combinations of BaP and S-9 concentrations**. *Indicates significance at 0.01 < *p* < 0.05, **indicates significance at *p* < 0.01. Bars are standard errors of three biological replicates.

In order to assess if BaP activation via S-9 influences toxicity in yeast, we performed wild type yeast growth curve assay at BaP concentrations of 0% IC_20_ (0 μM), 25% IC_20_ (82.5 μM), 50% IC_20_ (165 μM), and 100% IC_20_ (330 μM) with S-9 extract concentrations of 0, 1, and 2% by volume (Figure 4). Only the 165 μM BaP concentration showed statistically significant more growth inhibition with increasing S-9 extract concentrations (Figure 4). At 82.5 μM, we noted a very modest but significant decrease between the 1 and 2% S-9 but not between 0 and 1% nor 0 and 2%. No difference was seen at the highest BaP dose. Despite the ambiguity of the effect of S-9 on BaP toxicity, there was demonstrable statistical evidence for interaction between BAP and S-9 (*p*-value = 0.000323). In addition, while BAP toxicity is influenced by S-9 in a non-additive way, the significant effect on growth is sensitive to highly specific BAP and S-9 dose combinations, with maximal consequence centered at 165 μM BAP and 2% S-9 and resulting in an approximately 25% growth reduction relative to the control.

### Real-time protein expression in GFP-infused stress pathway ensemble yeast library

**Figure 5:**
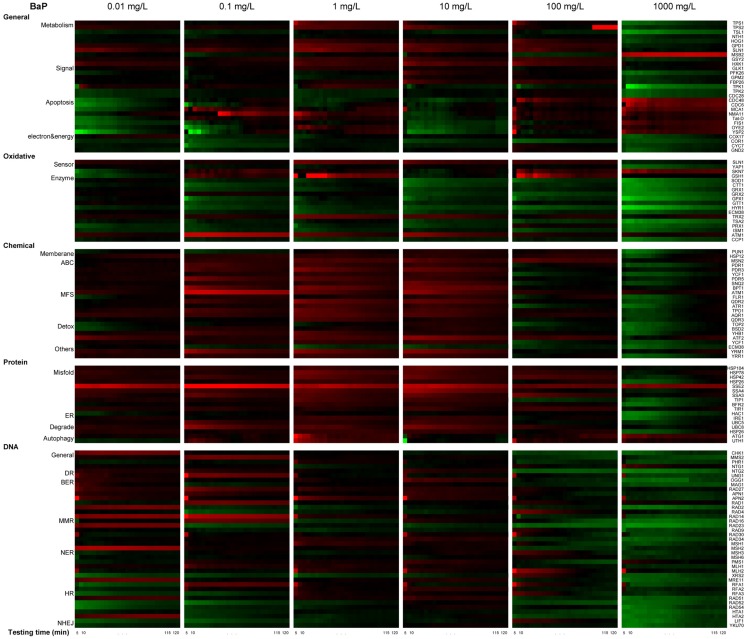
**Real-time protein expression profile of BaP exposure based on natural log of induction factor**. ln I as average of triplicate, Red spectrum colors indicate upregulation, green spectrum colors indicate downregulation in yeast. *X*-axis bottom: testing time, every 5 min for 2 h. *Y*-axis left: clusters of proteins by stress response pathways, *Y*-axis right: ORFs tested.

We examined real-time differential protein expression profiles (in comparison to control) of BaP-induced stress response over 2 h exposure times for six different concentrations (Figure 5).

**Figure 6:**
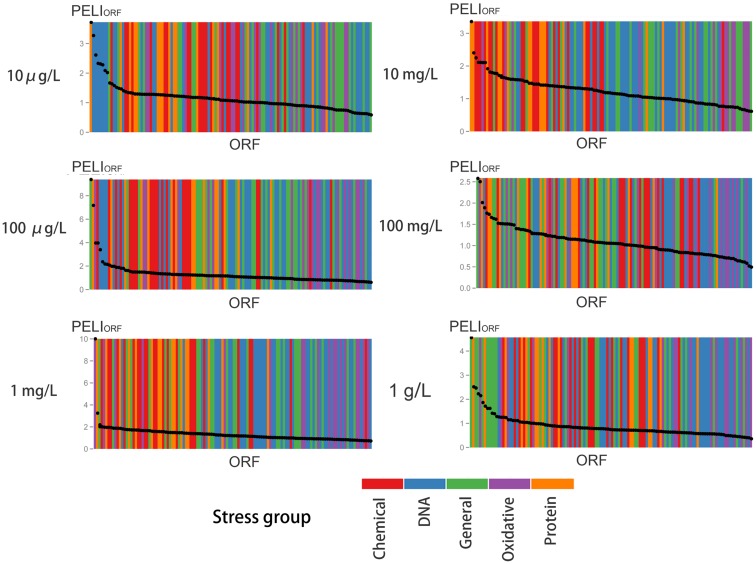
**Enrichment analysis of protein expression according to various stress groups for different concentrations of BaP exposure**. For each concentration, the ORFs are ranked in decreasing order by their PELI score (PELI_ORF_, average of triplicates). Different colors indicate different stress groups (see Table 1).

The profiles are consistent with a dose-dependent cellular response, with varying protein activation profiles and dynamic toxic responses at different dose concentrations. To further quantify and reveal the dominant stress response categories involved in exposure to different chemical concentrations, an expression level rank-based protein expression enrichment analysis was conducted and the results are shown in Figure 6. The proteins and associated stress categories that had higher expression change levels (ranking) were more clearly visualized, indicating that the cellular response shifted with concentration change, from DNA stress at low levels to chemical and protein stress at higher levels, and general stress response for the highest level. At relatively lower dose concentration (<0.1 mg/L), DNA damage repair pathways, primarily base excision repair, were activated. As the dose increases, stress response activation shifts to transport and membrane function, including a wide-ranging activation of the ABC and MFS transporters involved in chemical export. In addition, the protein stress response, including a series of heat shock proteins involved in recognition and repair of damaged/misfolded proteins, is activated. Finally, there is evidence of oxidative damage as indicated by activation of oxidant sensors and enzymes (*SLN1*, *TRX2*, and *GSH1*). At higher concentration (>100 mg/L), stress responses shifted to more general stress and were down regulated, while proteins for apoptosis increased, suggesting that cells transitioned from stress response to cell death.

According to the signal-to-noise ratio defined, 24 proteins were activated by a 10 mg/L BaP exposure and 14 proteins for 100 mg/L of BaP. An enrichment analysis was performed based on GO categories to identify significantly overrepresented categories among these activated ORFs, with the whole stress library selected as the reference in Table 6. The results suggested that the main biological process induced by a 10 mg/L BaP exposure focused on transmembrane transport and protein folding. Comparison of the activated proteins in the stress response GFP-tagged library with sensitive genes in the deletion library showed that there were few common proteins, possibly related to the difference in testing period (1G versus 5G) and the approach applied (protein expression versus gene deletion). Also, the GFP-tagged library only focused on stress response with limited ORFs tested (123 ORFs in the GFP-tagged library versus 4,757 ORFs in the gene deletion library), so there may a significant amount of sensitive genes not tested in the GFP-tagged library.

**Table 6:** **Overrepresented (*p*-value < 0.01) biological categories based on GO databases for a two hour exposure at 10 mg/L and 100 mg/L BaP in yeast (test set) with PELI_ORF_ > 1.5, with the stress library serving as a reference set**.

Category	*p*-value	Names of genes in category	Number of genes observed in category	Number of genes in category
**10 mg/L BaP EXPOSURE**				
**GO Molecular Function**
Unfolded protein binding [GO:0051082]	0.0033	*HSP104 HSP42 SSE2 SSA4 SSA3*	5	7

**GO Biological Process**
Transmembrane transport [GO:0055085]	1.20E−04	*ATM1 BPT1 QDR2 TPO1 AQR1 YRM1 HSP78 SSA4 SSA3*	9	14
Protein folding [GO:0006457]	2.10E−04	*HSP104 HSP78 HSP42 SSE2 SSA4 SSA3*	6	7
Transport [GO:0006810]	0.0025	*GLK1 TRX2 ATM1 SNQ2 BPT1 QDR2 TPO1 AQR1 YRM1 HSP78 SSA4 SSA3*	12	30
Protein refolding [GO:0042026]	0.007	*HSP104 HSP78 SSE2*	3	3
Intracellular protein transmembrane transport [GO:0065002]	0.007	*HSP78 SSA4 SSA3*	3	3
Establishment of localization [GO: GO:0051234]	0.007	*GLK1 TRX2 ATM1 SNQ2 BPT1 QDR2 TPO1 AQR1 YRM1 HSP78 SSA4 SSA3*	12	33

**GO Cellular Component**
Integral to membrane [GO:0016021]	0.0044	*SLN1 MSB2 ATM1 SNQ2 BPT1 QDR2 TPO1 AQR1 ATF2*	9	20
Intrinsic to membrane [GO:0031224]	0.001	*SLN1 MSB2 ATM1 SNQ2 BPT1 QDR2 TPO1 AQR1 ATF2*	9	22
**100 mg/L BaP EXPOSURE**				
**GO Biological Process**
Cytoskeleton organization [GO:0007010]	0.0090	*DC48 HSP42*	2	2

## Discussion

Although, BaP is a potent carcinogen, our understanding of its toxicity pathways is limited. Indeed, the toxicant can form DNA adducts and reactive oxygen species metabolites that are thought to be the main mechanisms of toxicity. However, these pathways do not fully account for the observed damage and it is likely that other pathways are concurrently at play. Moreover, since DNA adducts constitute only part of the parent BaP toxicity, the paucity of reliable biomarkers specific to the toxicant also motivates the identification of genes responsive to exposure. Although BaP toxicity was less in yeast than for human cells, this was consistent with similar prior studies involving benzene. Resistance to BaP seems to also increase with duration of exposure. Many of the pathways elicited were consistent with prior BaP toxicity studies, which identified reactive oxygen species and DNA damage, but our study also identified solute carriers and transmembrane transport as responsive to BaP exposure and as possible biomarkers. Moreover, addition of S-9 metabolic extract in addition to BaP had limited to no effect on the latter’s toxicity in *S. cerevisiae*.

### BaP less toxic to yeast than human cells

BaP was approximately 50–100 times less toxic in *S. cerevisiae* than has been observed in cultured human cell samples from individuals exposed to BaP (Aust et al., 1980; Vayssier-Taussat et al., 2001). Yeast lacks certain peroxidases found in humans, which are required to transform BaP metabolites, such as hydroquinone, to more toxic forms (North et al., 2011). Moreover, BaP causes human cell proliferation at low levels, largely depending on the proportion of metabolites produced, which could introduce variability in identifying the IC_20_ (Burchiel et al., 2007). In addition, although the BaP parent compound is relatively stable in the environment, the toxic metabolite(s) is (are) more reactive (Dabrowska et al., 2008). Nevertheless, the BaP IC_20_ is lower than was found for the three benzene metabolites hydroquinone, catechol, and 1,2,4-Benzentriol independently tested using the same method (North et al., 2011).

**Figure 7:**
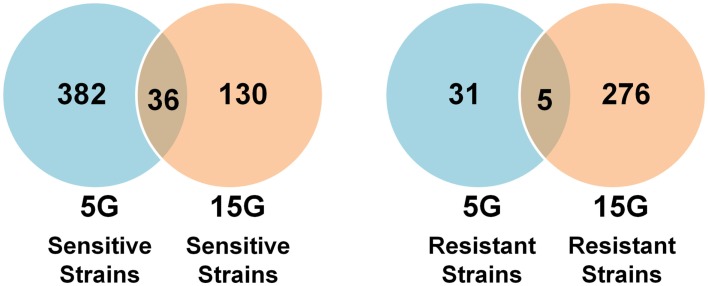
**Venn diagram overlap between sensitive and resistant strains across generations**.

We found more sensitive strains in the 5G assay and, conversely more resistant strains in the 15G exposure. Interestingly many of the strains that showed sensitivity in the 5G analysis were resistant in the 15G assays (Figure 7). Cellular responses may increase resistance in these yeast deletion strains over time. Indeed, there are relatively few strains in common between the 5G resistant and the 15G sensitive assays.

### Gene ontology and confirmations: Deletion and GFP-tagged library exhibit concordant mechanisms of toxicity and detoxification

Following from the enrichment analysis of the sensitive/resistant strains and activated ORFs, we identified four biological categories relevant to BaP toxicity. Namely, we found genes related to cellular oxidative stress, DNA damage and repair, and drug transmembrane transport and detoxification by export to be implicated in the response to BaP in both deletion and GFP-tagged libraries. While the toxicity pathways are similar to what has been identified in prior studies of BaP, the implication of transmembrane transport is novel and could prove useful in identifying future biomarkers of exposure.

#### Cellular oxidative stress and protein stress

BaP perturbs cellular redox homeostasis by producing quinones (BaP 6,12-quinone, BaP 6,12-hydroquinone, BaP 3,6-hydroquinone, BaP 3,6-quinone; Miller and Ramos, 2001). Further reduction of the quinones creates reactive oxygen species (ROS) in the form of superoxide and hydrogen peroxide (Miller and Ramos, 2001). From our results, we observe a perturbation of the cell redox homeostasis, as indicated by the sensitive deletion strains related to oxygen and radical detoxification (*PRX1*, *TRX2*, and *AHP1*), cellular redox homeostasis (*PRX1*, *TRX2*, *POR2*, *AHP1*, and *GLR1*), and enzymes necessary for glutathione – involved in mitigating oxidative stress – recycling (*OYE3*). It is important to note that the genes responsible for glutathione recycling were only significant in the 5G assay, indicating that NADPH recycling is more important in reducing oxidative stress for earlier generations, as was also found for the quinone metabolites of benzene in a prior yeast study (North et al., 2011). This is further confirmed by the activated oxidative stress ORFs in the GFP-tagged library, such as *SLN1* and *TRX2* in the 10 mg/L exposure, which imply that oxidative stress plays an important role in BaP toxicity. In addition, protein stress was observed in the GFP-tagged library as indicated by activation of protein folding and refolding categories for unfolded proteins such as *HSP104*, *SSE2*, *SSA4*, *UBC8*, etc., in five of the six concentrations. However, genes involved in response to protein misfolding were not observed in the deletion library, suggesting that the misfolding damage may be a temporary subsequent response of oxidative or chemical stress or that sufficient redundancy exists to mitigate the requirement for any particular gene product.

#### DNA damage and repair

Many of the confirmed deletion strains that exhibit sensitivity to BaP are associated with DNA damage and cell cycle disruptions that are concordant with observations in human *in vitro* studies. Indeed, in *S. cerevisiae*, *RAD6*, a ubiquitin-conjugating enzyme also involved in post-replicative repair, *RAD50*, implicated in the repair of double-stranded DNA breaks and homologous/non-homologous end-joining, *DNL4*, a DNA ligase for non-homologous end-joining, and *ELC1*, important in polyubiquitylating Rpb1 during global genome repair, were required for yeast tolerance to BaP (Jentsch et al., 1987; Chen et al., 2001; Hoege et al., 2002; Zhang and Paull, 2005; LeJeune et al., 2009). Non-homologous and homologous end-joining are known as the most important aspect of repairing BaP-induced DNA adducts in humans, so the significance of *DNL4* in yeast suggests some concordance in response to BaP (Natarajan and Palitti, 2008). Moreover, other genes in yeast appear to be important in responding to other types of DNA damage caused by ROS (e.g., hydroquinones) such as *DTD1*, involved in nonsense mutation suppression and *ELC1*, involved in global genomic repair (von der Haar and Tuite, 2007; LeJeune et al., 2009). While *SHM2* is important in yeast for synthesizing DNA bases and its homozygous deletion actually promotes resistance to most other toxicant surveyed in other studies, this was the reverse for BaP (Huang et al., 2008). Indeed, both direct and indirect BaP interference with DNA replication and repair are sources of cellular growth inhibition in yeast (Xie et al., 2003). In the GFP-tagged library, DNA stress response is stronger at lower concentrations, e.g., activation of double strand break repair (*RAD51* and *HTA2*) for a 10 μg/L exposure. For 10 mg/L, only base excision repair (*OGG1* and *APN2*) were activated for base damage repair related to base oxidation, suggesting the DNA damage induced by BaP at higher concentration focuses on bases, possibly related to oxidative damage.

#### Drug transmembrane transport and detoxification by export

Two yeast strains lacking drug transmembrane transport and detoxification by export, *qdr2*Δ, and *yrm1*Δ demonstrated significant growth inhibition in the presence of BaP. Both of these genes encode solute carriers (from family 22) and orthologs were noted to be upregulated in response to bisphenol A and benzo[a]pyrene in the livers of other organisms such as rats and mice as well as human cell cultures (Staal et al., 2006). Moreover, the expression of such genes has been proposed as a potential biomarker of exposure to chemicals and is an important response to chemical injury in human liver cells (Borlark, 2005). In both yeast and people, these solute carriers are specific to various organic anion molecules including glutathione, sulfate, and glucuronide conjugates, which are the ultimate products of BaP metabolism prior to excretion in humans (Pritchard et al., 2005). Interestingly, other solute carrier families have been implicated in human BaP toxicity, such as SLC38A5 which is thought to indirectly favor cis DNA adduct formation by increasing the chloride ion concentration thus catalyzing S_N_2 attack of the BPDE carbocation metabolite (Wolfe et al., 1994). However, literature concerning the role of solute family 22 in human BaP toxicity is limited and warrants further investigation given the potential role played by the orthologous gene in yeast BaP exposure and hydroquinone metabolite toxicity (Keum et al., 2006). Similarly, in the GFP-tagged assay, the two hour 10 mg/L exposure also demonstrated strong activation of proteins for detoxification by export, including transmembrane transport (such as *ATM1*, *BPT1*, *QDR2*, etc.) and transport (*GLK1*, *TRX2*, *ATM1*, etc.), which supports chemical export as a significant detoxification mechanism.

### Lack of growth response to S-9 metabolic adjuvant and BaP dose combinations

The combination of rat S-9 and BaP did not seem to alter growth inhibition in wild type yeast. Only the two percent by volume addition of rat S-9 had any significant effect, but this was only observed at the two lowest concentrations and only resulted in a maximum 1.3-fold increase in toxicity. Our results are consistent with the study by Hakura et al. (2001) that found a minimum concentration of approximately 1.85% S-9 by volume was needed to metabolize BaP. Indeed, 2% S-9 increased toxicity significantly more than would be expected from the 1% concentration, as illustrated by the statistical interaction between BAP and S-9 in our study. Rat S-9 is approximately 100 times more potent than S-9 reagents derived from human microsomes, so we would not expect human S-9 to increase the bioactivation of BaP and toxicity in yeast (Hakura et al., 2001). Very few studies have assayed the utility of S-9 in yeast; indeed, one study involving chlorinated ethylenes found no difference between treatments with and without S-9 (Koch et al., 1988). One possibility for the observed inconsistent effect of S-9 may be that the metabolites produced are more reactive and may not be able to effectively enter the yeast cells. The milieu (i.e., within yeast cells or in solution) where the BaP metabolism reaction occurs may also influence the amount and ratio of the metabolites produced and absorbed by yeast cells (Käppeli, 1986). Moreover, yeast cytochromes produced as part of the biosynthesis pathway for ergosterol, which is an important component of yeast membranes, have been shown to be able to metabolize exogenous chemicals as a side reaction (Alexander et al., 1974; Aoyama and Yoshida, 1978). Specifically, these enzymes are capable of oxidizing compounds to the toxic quinones and epoxides, which are products of BAP metabolism (Wiseman et al., 1978). In addition, these enzymes have also been shown to metabolize pro-carcinogens to their carcinogenic form (Callen et al., 1980). Consequently, while *S*. *cerevisiae* does not have the dedicated *bona fide* metabolism observed in higher organisms, some of its biosynthetic pathways may serve this purpose. Given our inconclusive results on the effect of S-9 on the toxicity of BAP, the known reactivity of the mammalian BAP metabolites, and the demonstrated metabolic action of endogenous yeast enzymes, S-9 may not be necessary for bioactivation in yeast, at least for this particular toxicant. It is also important to note, that the specific metabolites generated by the yeast system may not be identical to mammalian metabolites. Nevertheless, the statistically demonstrable interaction between BAP and S-9 suggests that using S-9 bioactivation, especially at concentrations of at least 2% by volume, may prove useful for other compounds whose independent toxicity is more subtle or ambiguous. Alternatively, anaerobic, high glucose concentrations (4–20%), inhibitors of mitochondrial protein synthesis, or respiration-impaired mutants have been shown to favor cytochrome production in *S. cerevisiae* and these conditions could also be used as part of a model for investigating other bio-activated toxicants (Wiseman and King, 1982).

### Human ortholog genes to yeast sensitive/resistant genes suggest possible biomarkers

From the sensitive and resistant genes, we identified orthologous human genes using the YOGY database (Table 7; Penkett et al., 2006). VDAC1 has been found to be involved in the cellular apoptotic response to BaP in mice, rats, and human cell lines (Huc et al., 2007; Andreau et al., 2012). Perez et al. (2008) did not find RAD50 expression to be upregulated by BaP exposure in normal human keratinocytes, but Brevik et al. (2012) determined that paternal exposure to BaP did up-regulate expression in mouse embryos. In this instance, RAD50 may serve as a biomarker of developmental toxicity and exposure to the toxicant. While SHMT2 has been implicated in tumor genesis in mice, no prior study has linked it to BaP exposure directly (Nilsson et al., 2012). Similarly, LIG4 upregulation has been associated with tobacco-related cancers and not BaP specifically, though BaP is a component of tobacco smoke (Wu et al., 2004). In addition, rodent studies of rats and mice found ortholog upregulation of UBE2B and PRDX5 as well as downregulation of DTD1 (Chen et al., 2001; van Kesteren et al., 2011). While none of the genes in Table 7, with the exception of select solute carriers previously discussed and VDAC1, have been conclusively linked to adverse outcomes in human cells and BaP exposure, they represent candidate susceptibility loci as they are related to carcinogenesis pathways.

**Table 7:** **Select yeast genes with significant response to BaP and their human orthologs**.

Yeast gene	Human gene	Name of human gene
*POR2*	VDAC1	Voltage-dependent anion-selective channel protein 1

*RAD50*	RAD50	DNA repair RAD50

*DNL4*	LIG4	DNA ligase 4

*RAD6*	UBE2B	Ubiquitin-conjugating enzyme E2B

*SHM2*	SHMT2	Serine hydroxymethyltransferase, mitochondrial

*DTD1*	C14orf126 (DTD1)	d-tyrosyl-tRNA(Tyr) deacylase 1

*AHP1*	PRDX5	Peroxiredoxin-5, mitochondrial

*GLR1*	GSR	Glutathione reductase

*QDR2*	SLC22A3, SLC22A6, SLC22A17, SLC22A12, SLC22A9, SLC22A1, SLC22A5, SLC22A11	Solute carriers

	SV2B	Synaptic vesicle protein

*TRX2*		Thioredoxin domain containing protein 3, 8, and 2 isoform 1; thioredoxin isoform 1

*PRX1*	PRDX6	Peroxiredoxin-6

*ELC1*	TCEB1	Transcription elongation factor B polypeptide 1

*CTT1*	CAT	Catalase

## Conflict of Interest Statement

The authors declare that the research was conducted in the absence of any commercial or financial relationships that could be construed as a potential conflict of interest.

## Supplementary Material

The Supplementary Material for this article can be found online at http://www.frontiersin.org/Toxicogenomics_/10.3389/fgene.2012.00316/abstract

## Footnotes

^1^http://www.geneontology.org/

^2^http://mips.helmholtz-muenchen.de/genre/proj/yeast/

^3^http://funspec.med.utoronto.ca/
